# A comparison of dosimetric parameters between tomotherapy and three-dimensional conformal radiotherapy in rectal cancer

**DOI:** 10.1186/1748-717X-8-181

**Published:** 2013-07-16

**Authors:** Mina Yu, Joo Hwan Lee, Hong Seok Jang, Dong Min Jeon, Jae Suk Cheon, Hyo Chun Lee, Jong Hoon Lee

**Affiliations:** 1 Department of Radiation Oncology, St. Vincent’s Hospital, College of Medicine, The Catholic University of Korea, 93-6 Ji-dong Paldal-gu, Suwon, Kyeonggi-do, Republic of Korea; 2 Department of Radiation Oncology, Seoul St. Mary’s Hospital, College of Medicine, The Catholic University of Korea, Seoul, Korea

**Keywords:** Conformal, Dosimetry, Preoperative radiotherapy, Rectal cancer, Tomotherapy

## Abstract

**Purpose:**

Tomotherapy for intensity-modulated radiation has been demonstrated to reduce unnecessary irradiations to adjacent organs at risk (OARs). The purpose of this study was to compare the dosimetric parameters between Tomotherapy and three-dimensional conformal radiotherapy (3D-CRT) in rectal cancer patients.

**Materials and methods:**

We redesigned three-dimensional conformal plans for 20 rectal cancer patients who had received short-course preoperative radiotherapy with Tomotherapy. The target coverage for 3D-CRT and Tomotherapy was evaluated with the following including the mean dose, V_nGy_, D_min_, D_max_, radiation conformality index (RCI), and radical dose homogeneity index (rDHI).

**Results:**

The mean PTV dose for Tomotherapy is significantly higher than that observed for the 3D-CRT (p = 0.043). However, there is no significant difference in the V_23.25Gy_, V_26.25Gy_, V_27.5Gy,_ and RCI values between Tomotherapy and 3D-CRT. However, the average rDHI (p < 0.001) value for Tomotherapy was significantly lower than that reported for the 3D-CRT. Tomotherapy significantly lowered the mean level of irradiation doses to the bladder, small bowel, and femur heads as compared to 3D-CRT.

**Conclusions:**

Tomotherapy could produce a favorable target coverage and significant dose reduction for the OARs at the expense of acceptable dose inhomogeneity of the PTV compared with 3D-CRT in rectal cancer patients.

## Introduction

According to the Korean National Cancer Screening Survey, the incidence of colorectal cancer has been increasing gradually in Korea. In addition, colorectal cancer is the third most common diagnosed malignancy, with an estimated 22,000 new cases per year [1]. Conventional treatment with fractionated radiation of 50.4 Gy per 28 fractions for six weeks is a standard regimen that has been widely accepted for positive therapeutic outcomes of locally advanced rectal cancer, which is based on results from prospective randomized trials [2,3]. Compared with postoperative radiotherapy, preoperative radiotherapy demonstrated an improvement in locoregional tumor control and resectability [2]. In European countries, a short-course radiation treatment regimen of 25 Gy per five fractions for a week has frequently been used due to the decreased expense and increased convenience of a five-day irradiation for rectal cancer patients as compared to a five-week schedule of conventionally fractionated irradiation [4,5]. A prospective randomized study has proven that there were no significant differences in survival and local tumor control between conventionally fractionated and short-course irradiation for locally advanced rectal cancer patients [6]. However, there have been several reports that short-course irradiation has negative side effects including the increased risk of late small bowel obstruction, necessitating hospital admission, and sexual dysfunction [7,8]. Therefore, clinicians have made considerable efforts to minimize the dose of irradiation to adjacent organs at risk (OARs) including the small bowel and bladder to avoid the long-term toxicity observed in rectal cancer patients.

Recently, techniques to improve the accuracy of radiation delivery to the target have advanced dramatically. Helical Tomotherapy (Accuary Inc., Sunnyvale, CA) which can involve image-guided radiation therapy using a megavoltage CT scan just prior to radiation treatment is one specific example of these advancements. Tomotherapy can also yield intensity-modulated radiation therapy (IMRT), which allows for highly conformal distributions of the dose of radiation to the target and minimizes the irradiation to the adjacent dose-limiting organs. Use of the IMRT technique to decrease an unnecessary irradiation to the bowel has been widely reported for the treatment of gynecologic cancers [9]. However, dosimetric studies comparing IMRT and three-dimensional conformal radiation therapy (3D-CRT) in rectal cancer patients are scarce and assessed only in a small series [10]. Thus, we compared the dosimetric parameters between Tomotherapy and 3D-CRT in rectal cancer patients who received preoperative radiotherapy**.**

## Methods

### Patients and simulation

Twenty consecutive patients with rectal cancer who had received short-course preoperative radiotherapy at Seoul St. Mary’s Hospital, Seoul, Korea from July 2010 to May 2011 were evaluated for the present study. They had locally advanced resectable disease (cT3 or cT4) with the inferior tumor margin located no further than 8 cm from the anal verge. The study participants included 14 men and 6 women, with a median age of 61 years (range, 39–77 years). This study was approved by the institutional review board of the Catholic University of Korea (KC10EIMS0025).

During treatment, patients were immobilized in the prone position using a foam cushion which covered the whole body. A contrast-enhanced CT was scanned for the treatment plan. Patients were instructed to have an empty bladder before the planning CT scan. The CT imaging ranged from the L1 vertebral body to 5 cm below the perineum, and axial images were obtained at 3-mm thickness and imported to the Pinnacle^3^ treatment planning system (Philips Radiation Oncology Systems, Fitchburg, WI).

### Treatment planning

Target volumes were defined according to the recommendation of the ICRU report 62 [11]. The clinical target volume included the gross tumor volume and the presacral, mesorectal, common and internal iliac lymph nodes. The planning target volume (PTV) was symmetrically generated with 3 to 5-mm margins around the clinical target volume. The OARs such as bladder, small bowel, and femur heads were contoured. The small bowel was outlined 3 cm above and below the PTV, and the bladder and femur heads were fully outlined.

**Figure 1:**
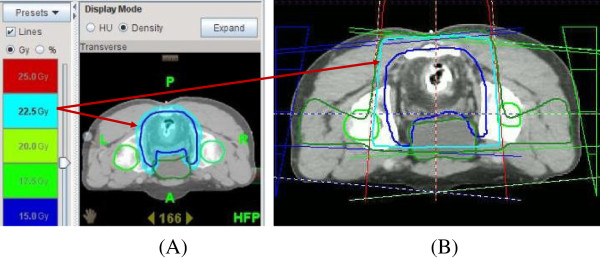
**A exhibits an intensity-modulated radiotherapy image with Tomotherapy, and B shows a three-field technique with three-diemnsional conformal radiation therapy.** Tomotherapy could avoid high-dose irradiation of 22.5 Gy to the bladder which is outlined in azure.

For the IMRT plan with Tomotherapy, the raw dosimetric data set of each patient was transferred from the Pinnacle^3^ treatment planning system to the Tomotherapy work-station (Figure 1A). For the 3D-CRT plan, a three-field technique (one anterior portal and two bilateral portals with wedge) was used (Figure 1B). Twenty-five Gy in five fractions delivered with 6 to 15 MV photon was prescribed for the PTV. Dose constraints for the PTV were as follows; (1) ≥ 98% of the PTV receives ≥ 95% of the prescribed dose, (2) ≤ 10% of the PTV receives ≥ 105% of the prescribed dose, (3) ≤ 5% of the PTV receives ≥ 110% of the prescribed dose. (4) None of the PTV receives ≥ 115% of the prescribed dose. No specific dose constraints for the OARs were used.

### Dosimetric evaluation

Dosimetric parameters to analyze target coverage and dose distribution in the PTV are as follows; (1) mean dose, (2) V_nGy_, percentage of the volume receiving radiation ≥ n Gy, (3) D_min_, minimum dose irradiated to the PTV, (4) D_max_, maximum dose irradiated to the PTV, (5) Radiation conformality index (RCI), PTV / V_95%_ (volume enclosed by the 95% of isodose line), and (6) Radical dose homogeneity index (rDHI): D_min_ / D_max_ in the PTV. The avoidance of irradiation to the bladder, small bowel, and femur heads was evaluated using the values such as mean dose and V_nGy_.

The dosimetric parameters of Tomotherapy and 3D-CRT were compared using the t-test and the difference was considered statistically significant at the *p* < 0.05 level.

## Consent

Written informed consent was obtained from the patient for publication of this report and any accompanying images.

## Results

### Dose distribution of the planning target volume (PTV)

The median of the PTV outlined in the 20 patients was 1076 cc (range, 796 to 1435 cc). The Tomotherapy and 3D-CRT plan met the prescription requirements for the PTV in all cases. Dose parameters for the PTV in the Tomtherapy and 3D-CRT plan were listed and compared in Table 1. The mean PTV dose for Tomotherapy is significantly higher than that for the 3D-CRT (25.58 ± 0.35 vs. 25.19 ± 0.74 Gy, *p* = 0.043). However, there were no significant differences in the V_23.25Gy_, V_26.25Gy_, and V_27.5Gy_ values between the Tomotherapy and 3D-CRT. Average D_max_ values for the Tomotherapy and 3D-CRT were not significantly different. However, the average D_min_ value for the Tomotherpay was significantly lower than that for the 3D-CRT (17.97 ± 1.93 vs. 22.05 ± 2.76 Gy, *p* < 0.001), and average rDHI value for the Tomotherapy is significantly lower than that for the 3D-CRT plan (0.70 ± 0.01 vs. 0.83 ± 0.04, *p* < 0.001). The average RCI values for the Tomotherapy and 3D-CRT were 1.01 and 1.00, respectively, and there is no significant difference in the average RCI value between the two modalities.

**Table 1:** Comparison of dose parameters for the planning target volume between Tomotherapy and 3-dimensional conformal radiation therapy

**Parameters**	**Tomotherapy**	**3D-CRT**	***p *value**
Mean dose (Gy)	25.58 ± 0.35	25.19 ± 0.74	0.043
V_23.25Gy_ (%)	99.45 ± 0.57	99.37 ± 0.86	0.225
V_26.25Gy_ (%)	10.03 ± 25.66	10.28 ± 7.52	0.485
V_27.5Gy_ (%)	0.26 ± 0.87	0.10 ± 0.12	0.414
D_min_ (Gy)	17.97 ± 1.93	22.05 ± 2.76	< 0.001
D_max_ (Gy)	27.28 ± 0.73	27.18 ± 0.38	0.588
RCI	1.01 ± 0.00	1.00 ± 0.00	0.120
rDHI	0.70 ± 0.01	0.83 ± 0.04	< 0.001

*RCI* radiation conformality index.

*rDHI* radical dose homogeneity index.

*D*_*min*_ minimum dose irradiated to the planning target volume.

*D*_*max*_ maximum dose irradiated to the planning target volume.

*V*_*nGy*_ percentage of the volume receiving radiation ≥ n Gy.

*3D-CRT* 3-dimensional conformal radiation therapy.

### Avoidance of organs at risk (OARs)

Table 2 shows the mean irradiation doses of the OARs including the bladder, small bowel, and femur heads. The mean doses for the bladder, small bowel, and femur heads with the Tomotherapy were significantly lower than those reported for the 3D-CRT.

**Table 2:** Comparison of irradiation doses to the organs at risk between Tomotherapy and 3-dimensional conformal radiation therapy

**Organs at risk**	**Tomotherapy, mean (Gy)**	**3D-CRT, mean (Gy)**	***p *value**
Small bowel	8.25 ± 2.24	12.34 ± 3.69	< 0.001
Bladder	14.95 ± 2.93	20.15 ± 2.13	< 0.001
Right femur head	11.05 ± 1.74	17.72 ± 1.46	< 0.001
Left femur head	10.80 ± 1.75	17.74 ± 1.38	< 0.001

*3D-CRT* 3-dimensional conformal radiation therapy.

**Figure 2:**
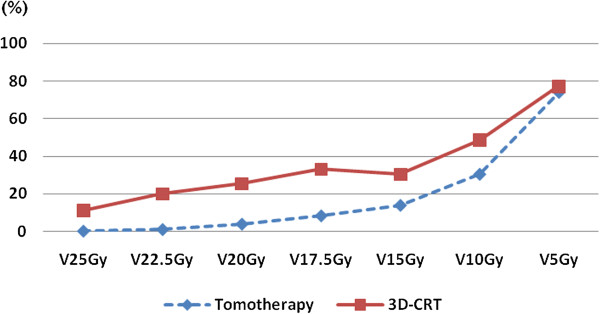
**Tomotherapy significantly reduced the V**_**25Gy**_**, V**_**22.5Gy, **_**V**_**20Gy**_**, V**_**17.5Gy **_**, V**_**15Gy**_**, and V**_**10Gy **_**value of the small bowel as compared to 3D-CRT.** However, the V_5Gy_ value which had been associated with the low irradiation range of the normal tissue was not significantly different between the two modalities.

We investigated the dosimetric parameters of the small bowel, which is the most critical organ in the rectal cancer patients who received the preoperative pelvic irradiation. Table 3 summarizes the V_25Gy_, V_22.5Gy,_ V_20Gy_, V_17.5Gy_ , V_15Gy_, V_10Gy,_ and V_5Gy_ values of the small bowel with Tomotherapy and 3D-CRT. Tomotherapy produced significantly lower V_25Gy_, V_22.5Gy,_ V_20Gy_, V_17.5Gy_ , V_15Gy_, and V_10Gy_ values for the small bowel as compared to the 3D-CRT. However, the V_5Gy_ value, which reflected the low irradiation range of the normal tissue, was not significantly different between Tomotherapy and 3D-CRT (Figure 2).

**Table 3:** Comparison of dose parameters for the small bowel between Tomotherapy and 3-dimensional conformal radiation therapy

**Values**	**Tomotherapy, mean (Gy)**	**3D-CRT, mean (Gy)**	***p *value**
V_25Gy_ (%)	0.10 ± 0.24	10.92 ± 12.63	< 0.001
V_22.5Gy_ (%)	0.93 ± 1.10	19.88 ± 15.73	< 0.001
V_20Gy_ (%)	3.79 ± 6.23	25.32 ± 16.44	< 0.001
V_17.5Gy_ (%)	8.19 ± 12.06	33.10 ± 18.71	< 0.001
V_15Gy_ (%)	13.79 ± 20.65	30.28 ± 19.16	< 0.001
V_10Gy_ (%)	30.28 ± 13.97	48.55 ± 19.03	0.001
V_5Gy_ (%)	73.74 ± 22.82	77.30 ± 15.64	0.568

*V*_*nGy*_ percentage of the volume receiving radiation ≥ n Gy.

*3D-CRT* 3-dimensional conformal radiation therapy.

## Discussion

IMRT modulates the beam intensity in several portals and has been demonstrated to achieve an effective coverage of the target tissue while avoiding damage to the normal tissue during treatment. Recently, IMRT has established an important role for the treatment of several malignancies through the promise of excellent local control of disease and reducing the potential complications inherent to external irradiation. Luxton G *et al.* reported that the accurate delivery of IMRT for prostate cancer could limit the dose to normal tissues and allow for a reduction in the rate of complication while maintaining the probability of tumor control [12]. A report by Bazan JG *et al.* indicated that IMRT was associated with less gastrointestinal, dermatologic, and hematologic toxicities, reduced need for treatment breaks, and excellent tumor control, compared with 3D-CRT in patients with anal cancer [13]. The National Comprehensive Consensus Network guidelines also recommend IMRT for the treatment of anal cancer patients in addition to 3D-CRT [14].

In rectal cancer patients, the preoperative short-course pelvic irradiation has demonstrated a definitive efficacy in reducing the local failure rate, compared with the rates reported for surgery alone [4,5]. However, it has given rise to the incidence of late small bowel morbidities such as intestinal obstruction and chronic diarrhea that could alter the therapeutic ratio [7,8]. Prognostic factors that increase the risk of late small bowel complications include extended fields out of the pelvis, irradiation dose, inappropriate irradiation technique, and increased small bowel irradiated volumes [15]. The irradiation dose and volume of the small bowel in rectal cancer patients are of major concern to clinicians [16]. Thus, we evaluated the dosimetric parameters of IMRT with Tomotherapy in rectal cancer patients. We hypothesized that Tomotherpay could reduce the overall dose to the normal tissue while maintaining the dose to the target, as compared to treatment with 3D-CRT.

In our study, the mean PTV dose for Tomotherapy is significantly higher than that for the 3D-CRT (25.58 ± 0.35 vs. 25.19 ± 0.74 Gy, *p* = 0.043) while Tomotherapy maintaining ≥ 98% of the PTV receives ≥ 95% of the prescribed dose and ≤ 10% of the PTV receives ≥ 105% of the prescribed dose. However, the rDHI for Tomotherapy is significantly lower than that for the 3D-CRT (0.70 ± 0.01 vs. 0.83 ± 0.04, *p* < 0.001) since the average D_min_ value for the Tomotherpay was significantly lower than that for the 3D-CRT (17.97 ± 1.93 vs. 22.05 ± 2.76 Gy, *p* < 0.001). Local tumor control is related to the mean dose rather than to the minimum dose in the target [17]. Moreover, a modest number of cold spots are not related to the reduced tumor control, and it is important to know the location and volume of the cold spots [18]. When we have checked our Tomotherapy plans to identify the location of cold spots, they were very small and were predominantly located in the small bowel region adjacent to the PTV, which had a low probability of tumor involvement.

In our analysis, Tomotherapy significantly reduced the mean dose irradiated to the OARs such as bladder, small bowel, and femur heads as compared to 3D-CRT. Specifically, Tomotherapy significantly reduced the high to intermediate irradiation range (V_25Gy_, V_22.5Gy,_ V_20Gy_, V_17.5Gy_, V_15Gy_, and V_10Gy)_ to the small bowel compared to 3D-CRT. However, the V_5Gy_ values which meant by low irradiation range of the radiation dose were similar between the Tomotherapy and 3D-CRT.

The result of the IMRT plans was associated with a trade-off between the coverage of the target and avoidance of the OSRs. We could attain optimal Tomotherapy plans, which significantly reduced the mean irradiation dose to the OARs at the expense of acceptable dose inhomogeneity of the PTV, because the goal of our IMRT plans was sparing of the OARs rather than full coverage of the target. Even though our study had inherent limitations including the retrospective nature and small patient number, our results support further investigation of IMRT for the treatment of rectal cancer patients [19-22].

In conclusion, our study shows that the use of Tomtherapy could produce favorable coverage for the target and decrease the mean dose for the OARs as compared to 3D-CRT for treatment of patients with rectal cancer. For a definitive conclusion, the Tomotherapy technique should be tested and compared with conventional radiation in a prospective series.

## Competing interests

All authors declare that they have no competing interests.

## Authors’ contributions

JHL: idea and concept. DMJ, JSC, and HSJ: design and development of Study. JHL, HCL, and HSJ: statistical analysis. JHL and MY: writing of manuscript and study coordinator. JHL and HSJ: final revision of manuscript. All authors read and approved the final manuscript.
